# Microbiome network remodeling is associated with soil lipid metabolism in safflower under different cropping system-location combinations: an omics-based dissection

**DOI:** 10.3389/fmicb.2026.1898896

**Published:** 2026-09-07

**Authors:** Yue Yang, Weijia Jia, Jin Xie, Jinliang Qiu, Chunhua Li, Chenxi He, Mingjin Luo, Lin Zhao, Yanwei Wang

**Affiliations:** 1 College of Construction Engineering, Yunnan Agricultural University, Kunming, Yunnan, China; 2 Department of Art, Tangshan University, Tangshan, Hebei, China; 3 College of Tea Science, Yunnan Agricultural University, Kunming, Yunnan, China; 4 Cash Crop Workstation of Lijiang, Lijiang, Yunnan, China

**Keywords:** metabolomics analysis, microbial community, rotation, safflower, soil lipid metabolism, understory planting

## Abstract

This study investigated differences in safflower rhizosphere soil microbiota and metabolic functions among three planting pattern-location combinations (PLMEs): soybean-safflower rotation at Chenghai (CH), tobacco-safflower rotation at Longpan (ZYHY), and apple orchard intercropping at Lijiang (LJ). Each PLME comprised 6 plots. Bacterial α-diversity was significantly higher in the CH system, whereas fungal α-diversity peaked under the ZYHY system. Correlation analysis showed that bacterial α-diversity was negatively correlated with available potassium, while fungal α-diversity was negatively correlated with catalase activity. Microbial community structures significantly varied among the different PLMEs, with redundancy analysis indicating that bacterial variation was mainly explained by electrical conductivity, while fungal variation was mainly explained by available phosphorus. Under the adopted network-construction procedure, sample-specific subnetwork analysis suggested that the bacterial subnetworks in CH were more topologically complex and stable, while the fungal subnetworks in ZYHY were more complex than those in other treatments; no marked differences in fungal subnetwork stability were observed. These findings remain exploratory. Although dominant microbial taxa were unchanged, their relative abundances varied notably. Non-targeted metabolomics analysis identified significant shifts in glycerophospholipid metabolism, with five key metabolites, including L-serine, phosphatidylethanolamine, and lecithin, serving as biomarkers strongly correlated with genera such as *Gaiella, Microlunatus,* and *Mortierella*. An exploratory structural equation modeling analysis revealed significant positive associations of bacterial α-diversity and bacterial network complexity with glycerophospholipid metabolism, and a significant negative association of fungal network complexity with glycerophospholipid metabolism. Overall, our results suggest that the CH system is associated with higher bacterial diversity, greater microbial network stability, and alterations in key metabolic pathways. Future studies involving direct measurements of plant growth, yield, and disease incidence are required to validate whether such rhizosphere changes are associated with agronomic benefits.

## 1 Introduction

*Carthamus tinctorius* L. (safflower) is an annual herbaceous dicotyledon in the genus *Carthamus* of the family Compositae (Asteraceae), and is also known as honghua cai, cao honghua, or hong lanhua. Its petals, filaments, and seeds are all used for food and medicine, making it an important medicinal plant in China (Zhou et al., 2014; Xian et al., 2022). At present, safflower production predominantly follows a monocropping pattern (Zanetti et al., 2022). However, continuous monoculture leads to many problems, including soil quality decline, biodiversity loss, and frequent outbreaks of pests, weeds, and diseases such as root rot, which often result in substantial yield reductions (Adams et al., 2025). Consequently, the associations between farmland biodiversity restoration and the artificial cultivation environment, growth characteristics, and secondary metabolite accumulation of safflower have become a research hotspot (Bose et al., 2023; Liu C. Q. et al., 2024). Recently, various planting models – including intercropping, crop rotation, and under-forest cultivation – have gradually emerged and been applied to overcome the obstacles caused by monoculture of traditional Chinese medicinal plants (Xu et al., 2021; Li et al., 2025; Sun et al., 2022), and have become a frontier strategy for sustainable land management. These planting patterns, shaped by years of experience with specific plant combinations and local site conditions (rather than existing independently), have collectively been associated with soil properties and microbial communities.

Soil microorganisms are central to soil ecosystem functions, directly influencing soil quality, fertility, and plant health (Rakhmatova et al., 2026). Within the rhizosphere, the composition and abundance of microbial communities – particularly bacteria and fungi – play critical and distinct roles (You et al., 2025). For instance, certain bacterial taxa (e.g., *Burkholderia* species) can solubilize phosphate and produce plant growth-promoting hormones; *Rhizobium* species are well known for biological nitrogen fixation; and many saprotrophic fungi (members of Basidiomycota) are efficient decomposers of recalcitrant organic matter such as lignin and cellulose (Zholdasbek et al., 2026; Haq et al., 2025). These functional roles, however, are context-dependent and vary with planting patterns. For example, continuous monoculture of Yongfeng yam reduces soil pH and increases available phosphorus (AP) and potassium (AK), thereby reducing bacterial diversity and altering bacterial composition. Beneficial bacterial taxa (e.g., Proteobacteria and Firmicutes) are depleted, while pathogenic taxa (e.g., *Bacillus* species) are enriched, ultimately affecting yam yield and quality (Yao et al., 2023). By contrast, rotating soybean with maize or wheat has been associated with the accumulation of soil organic matter (OM), AP, copper, and manganese, with higher abundance of beneficial bacteria (*Tausonia* and *Mortierella* species), with bacterial community diversity and structure, and with fewer problems related to continuous monoculture (Zhang et al., 2024).

Variations in soil microbial diversity, community composition, and metabolite composition are associated with the growth of medicinal plants, and different planting models are associated with changes in the rhizosphere microecology of medicinal plants (Zhao et al., 2025; Shao et al., 2025). However, research on how safflower planting patterns affect soil properties, microorganisms, and metabolite profiles remains scarce. Therefore, based on the Illumina MiSeq second-generation high-throughput sequencing platform, this study established three PLMEs: soybean–safflower rotation (at Chenghai), tobacco–safflower rotation (at Longpan), and under-apple-orchard cultivation (at Lijiang). Using microbial sequencing and metabolomics technologies, we analyzed the composition of microorganisms and soil metabolites, while also measuring soil physicochemical properties. The objectives of this research were twofold: (1) to characterize differences in diversity, community structure, networks, and metabolism of safflower rhizosphere soil microorganisms among the three PLMEs; and (2) to compare, from the perspective of sustainable land use, the associations of each PLME with soil microbial and metabolic traits. This study provides correlative observations pertaining to the ecological cultivation of safflower, which may have reference value for medicinal plant cultivation, land-use efficiency, and economic outcomes.

## 2 Materials and methods

### 2.1 Plant and soil materials

The research sites were located in Chenghai Town (100°38′–100°41′E, 26°17′–26°28′N, altitude 1,500 m), Longpan Township (99°39′–100°01′E, 26°17′–26°28′N, altitude 1850 m), and Gucheng District (100°25′E, 26°52′N, altitude 2,400 m) of Lijiang City, Yunnan Province, China. The specific details of the research sites are as follows:

**Table:** 

Site characteristics	Chenghai town experimental site	Longpan township experimental site	Gucheng district experimental site
Administrative Region	Central Yongsheng County, Lijiang City, Yunnan Province	Northwest of Yulong Naxi Autonomous County, Lijiang City, Yunnan Province	Gucheng District, Lijiang City, Yunnan Province
Climate Zone	Mid-subtropical plateau monsoon climate zone (Jinsha River dry-hot valley)	South temperate low-latitude plateau mountain monsoon climate zone	South temperate low-latitude plateau mountain monsoon climate zone
Mean Annual Temperature	19.2 °C	13.2 °C	12.79 °C
Temperature Range	Max 34.0 °C, Min 2.0 °C	Max 32.3°C, Min −6.0 °C	Hottest month (Jul) 17.9 °C, Coldest month (Jan) 5.9 °C
Annual Sunshine Hours	2,700 h	2463.4 h	2,530 h
Annual Rainfall	699.3 mm	968.3 mm	935 mm
Rainfall Pattern	Rainfall concentrated from May to October, distinct dry and wet seasons	Rainfall concentrated from June to September, distinct dry and wet seasons	Rainy season (Jun–Oct) accounts for >90% of annual rainfall; dry season (Nov–May) has little rain
Annual Evaporation	2,169 mm	~1800 mm	~1,600 mm
Rotation/Previous Crop	Rotated with soybeans	Rotated with tobacco	No previous crops; planted under apple trees

Because each PLME was established at a distinct geographical site (Chenghai, Longpan, or Lijiang), the cropping system and location are confounded in this study design. Therefore, all results are interpreted as differences among the three PLMEs as a whole, rather than as associations with the cropping system alone.

Each study area covered 25 acres. From each area, six sampling plots (10 m × 10 m, with at least 5 m spacing between plots) were randomly selected and marked accordingly for specimen collection. Sampling was conducted in late May 2024. Soil samples were collected at 10:00 a.m. on a sunny morning during the peak flowering period of safflower, ensuring that the plants at all three experimental sites were at the same physiological stage. Within each plot, five sampling points were established: one at the center and the other four at the corners, at least 0.5 m from the plot edges. At each sampling point, three safflower plants were randomly selected, and their entire root systems were carefully excavated with a stainless-steel shovel. Only healthy, disease-free plants with intact root systems were chosen. After gently shaking off loosely attached soil, the soil firmly adhering to the roots (within 0–2 mm) was collected with a sterile brush and designated as rhizosphere soil (Lundberg et al., 2012). A total of 15 plants (3 per sampling point × 5 points) were collected and combined to form one sample representing each plot. Thus, across the three experimental sites, with six replicate plots per site, a total of 18 rhizosphere soil samples were collected. Fresh soil samples were first passed through a 2-mm sieve. Half of each soil sample was stored at 4 °C for physicochemical analysis, while the other half was stored at −80 °C for microbial sequencing and metabolite analysis.

### 2.2 Determination of soil physicochemical parameters

At least 100 g of fresh soil was sampled and air-dried naturally at room temperature for subsequent determination of electrical conductivity (EC), pH, AP, and AK. The remaining fresh soil sample was used to determine the activities of urease (SUE), alkaline phosphatase (ALP), and catalase (CAT), as well as the contents of alkali-hydrolyzable nitrogen (HN), ammonium nitrogen (NH₄^+^-N), and nitrate nitrogen (NO₃^−^-N). Detailed analytical methods and instruments are described in Supplementary material 2.1.

### 2.3 16S and ITS gene sequencing analysis

After extracting microbial DNA from safflower rhizosphere soil, the V3–V4 area of the bacterial 16S rRNA gene and the fungal ITS area were enlarged using specific primers. Purification was executed on the PCR products. Then, they were quantified and sequenced on the Illumina MiSeq platform. The resulting raw sequences underwent quality control (QC), assembly, and clustering into operational taxonomic units (OTUs), followed by taxonomic annotation. Finally, the sequences were normalized for subsequent analysis. The sequences have been uploaded to the NCBI Sequence Read Archive under accession numbers (PRJNA1333617) and (PRJNA1334284). Detailed experimental procedures and parameters are provided in Supplementary material 2.2.

### 2.4 Metabolite detection and analysis

Metabolites were extracted from soil samples using a methanol–water solution and analyzed by LC–MS/MS. The initial data were preprocessed with Progenesis QI software and matched against the HMDB, Metlin, and MJDB databases for metabolite identification. All data were normalized prior to subsequent analysis (Dai and Sun, 2022). The raw metabolomics data have been deposited in the MetaboLights repository^1^ with the identifier MTBLS15129. Detailed extraction and analytical parameters are provided in Supplementary material 2.3.

### 2.5 Construction of microbial co-occurrence networks

Global co-occurrence networks were first constructed based on the top 50 most abundant bacterial and fungal genera across all samples. Significant pairwise associations between genera were identified using Spearman correlation coefficients, and the networks were visualized with Gephi. For each sample, subnetworks were extracted from the global networks using the ‘igraph’ R package. Topological coefficients were calculated for each sample subnetwork to estimate the network complexity. Robustness was computed on the global networks using a base R function (Xu et al., 2023). As complementary evidence, positive and negative cohesion were calculated for the six replicate subnetworks per treatment using ‘igraph’, and the absolute ratio of negative to positive cohesion was used as an additional indicator of network stability (De Vries et al., 2018). Detailed filtering thresholds, computational methods, and definitions of all topological metrics are provided in Supplementary material 2.4. These subnetworks and their derived metrics depend on the chosen network-construction parameters and should be interpreted as exploratory.

### 2.6 Statistical analysis

Microbial community characteristics were evaluated using diversity indices and principal coordinate analysis, with between-group differences compared by statistical tests. Correlation analysis, and exploratory structural equation modeling was used to examine the associations among soil physicochemical characteristics, microbial community attributes, and metabolites. Detailed analytical methods, software tools, and model parameters are provided in Supplementary material 2.5.

## 3 Results

### 3.1 Soil physicochemical properties and microbial diversity in the safflower rhizosphere differ among PLMEs

**Figure 1:**
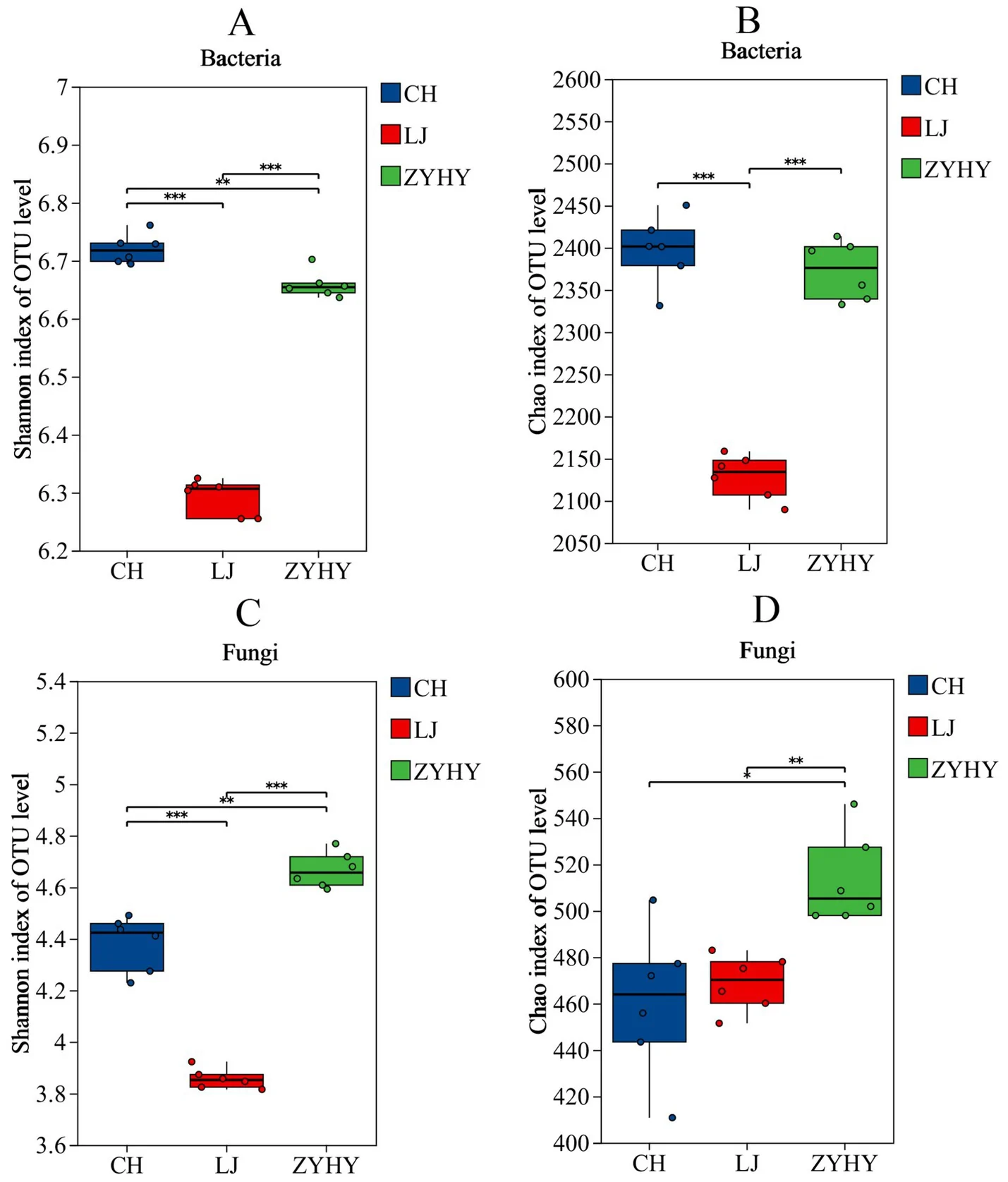
Bacterial **(A, B)** and fungal **(C, D)** α-diversity (Shannon index and Chao index) of safflower rhizosphere soil from the three PLMEs. Significance levels: ***p < 0.001, **p < 0.01, *p < 0.05, *p-*values were adjusted for multiple testing using the Benjamini–Hochberg (BH) method.

Following QC, 1,289,142 and 1,246,184 valid sequences were obtained for 16S rRNA and ITS sequencing, respectively. At a 97% similarity level, OTU clustering and rarefaction resulted in 45,518 bacterial OTUs and 54,952 fungal OTUs. Based on the OTU level, α-diversity indices of soil microorganisms (Shannon index and Chao index) were calculated. The results showed that among the safflower rhizosphere soil samples under different planting patterns (Figure 1), soybean–safflower rotation in Chenghai (CH; safflower rhizosphere soil samples with soybean as the preceding crop) had the highest bacterial α-diversity index, tobacco–safflower rotation in Longpan (ZYHY; safflower rhizosphere soil samples with tobacco as the preceding crop) had the highest fungal α-diversity index, and safflower under apple orchard in Lijiang (LJ; safflower rhizosphere soil samples cultivated under apple orchards) had the lowest bacterial and fungal α-diversity indices. As shown by correlation analysis (Table 1), the soil physicochemical characteristics most tightly correlated with bacterial α-diversity were HN, AP, and AK (negative), while those most closely associated with fungal α-diversity were EC (positive) and CAT (negative).

**Table 1:** Pearson correlation between bacterial and fungal α-diversity and soil physicochemical factors.

Index	pH	EC	SUE	ALP	CAT	HN	NH_4_^+^-N	NO_3_^,^ N	AP	AK
B-S	−0.511*	0.212	−0.837**	−0.722**	−0.846**	−0.952**	−0.804**	0.003	−0.921**	−0.984**
B-C	−0.544*	0.265	−0.791**	−0.678**	−0.869**	−0.920**	−0.757**	0.001	−0.881**	−0.952**
F-S	−0.637*	0.625*	−0.498	−0.318	−0.872**	−0.713**	−0.480	0.391	−0.635*	−0.806**
F-C	−0.320	0.548**	0.713	−0.363	−0.478	−0.120	−0.229	−0.545*	0.091	−0.095

Significance levels: *p_FDR_< 0.05; **p_FDR_ < 0.01. *p*-values were adjusted for multiple testing using the Benjamini–Hochberg (BH) method.

### 3.2 Analysis of rhizosphere soil microbial community structure of safflower under different PLMEs

**Figure 2:**
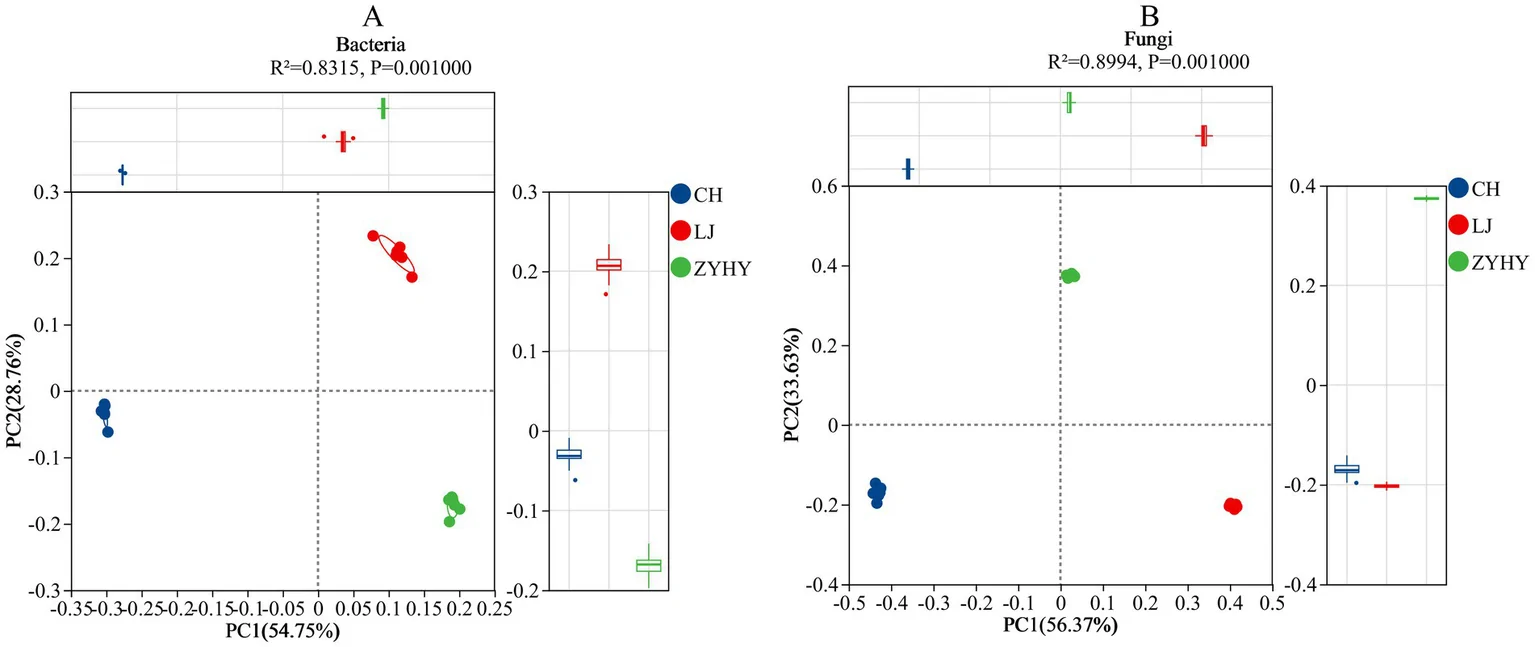
Principal coordinates analysis (PCoA) based on OTU level showing differences in bacterial **(A)** and fungal **(B)** community composition in soil samples. Group differences were tested by PERMANOVA (999 permutations).

**Figure 3:**
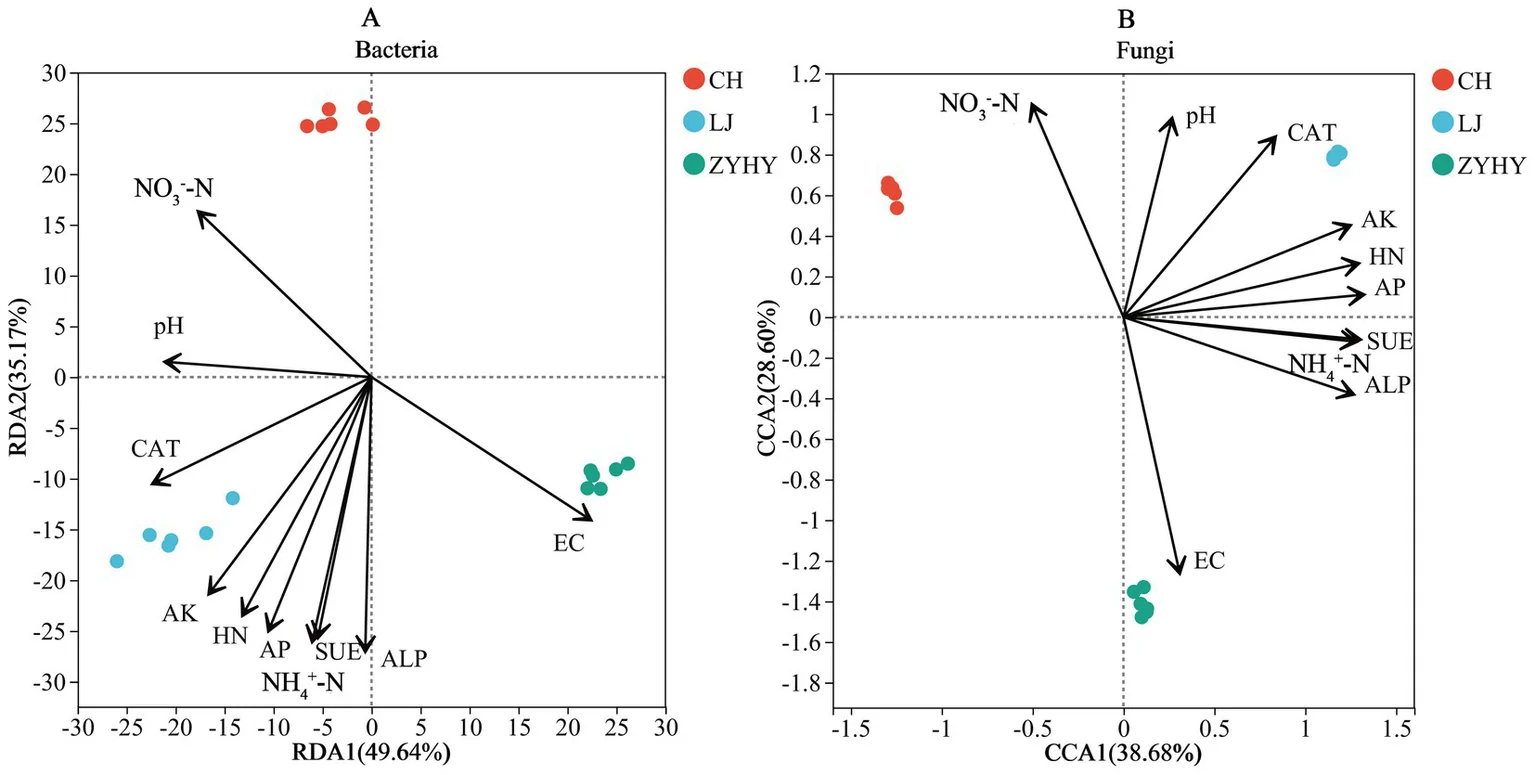
Redundancy and canonical correspondence analyses showing the relationships between environmental factors and bacterial **(A)** and fungal **(B)** communities at the OTU level.

Principal coordinates analysis (PCoA) results indicated that the bacterial community composition (*R*^2^ = 0.8315, *p* = 0.001) and fungal community composition (*R*^2^ = 0.8994, *p* = 0.001) of the three groups were significantly separated (Figure 2). PERMDISP (permutational analysis of multivariate dispersions) tests were subsequently performed. Both bacterial (*p* = 0.351) and fungal (*p* = 0.319) *p*-values exceeded 0.05, indicating that no statistically significant differences in multivariate dispersion were detected (Supplementary Table S2). Detrended correspondence analysis yielded maximal gradient lengths of 2.183 for the bacterial community and 4.320 for the fungal community; accordingly, redundancy analysis and canonical correspondence analysis were conducted for the bacterial and fungal communities, respectively (Figure 3). The findings revealed that bacterial community composition was most strongly associated with EC and AK, whereas fungal community composition was most strongly associated with AP and EC (Table 2).

**Table 2:** Explained variance (Explains) and *p* values between environmental factors and microbes following redundancy analysis.

Microbial beta diversity	Statistic	pH	EC	SUE	ALP	CAT	HN	NH_4_^+^-N	NO_3_^−^-N	AP	AK
β_B_	E	0.012	**0.431**	0.007	0.010	0.008	0.013	0.028	0.009	0.007	**0.389**
	*p*_FDR_	0.613	**0.010**	0.756	0.704	0.756	0.436	0.072	0.704	0.756	**0.010**
β_F_	E	0.029	**0.276**	0.029	0.037	0.026	0.020	0.013	0.032	**0.385**	0.016
	*p*_FDR_	0.280	**0.010**	0.427	0.140	0.436	0.689	0.756	0.280	**0.010**	0.756

The numbers in bold indicate remarkable disparities at *p* < 0.05 (*β*_B_, bacterial community structure; *β*_F_, fungal community structure). *p*_FDR_values were adjusted for multiple testing using the Benjamini–Hochberg (BH) method.

### 3.3 Co-occurrence network analysis of microbial communities

**Figure 4:**
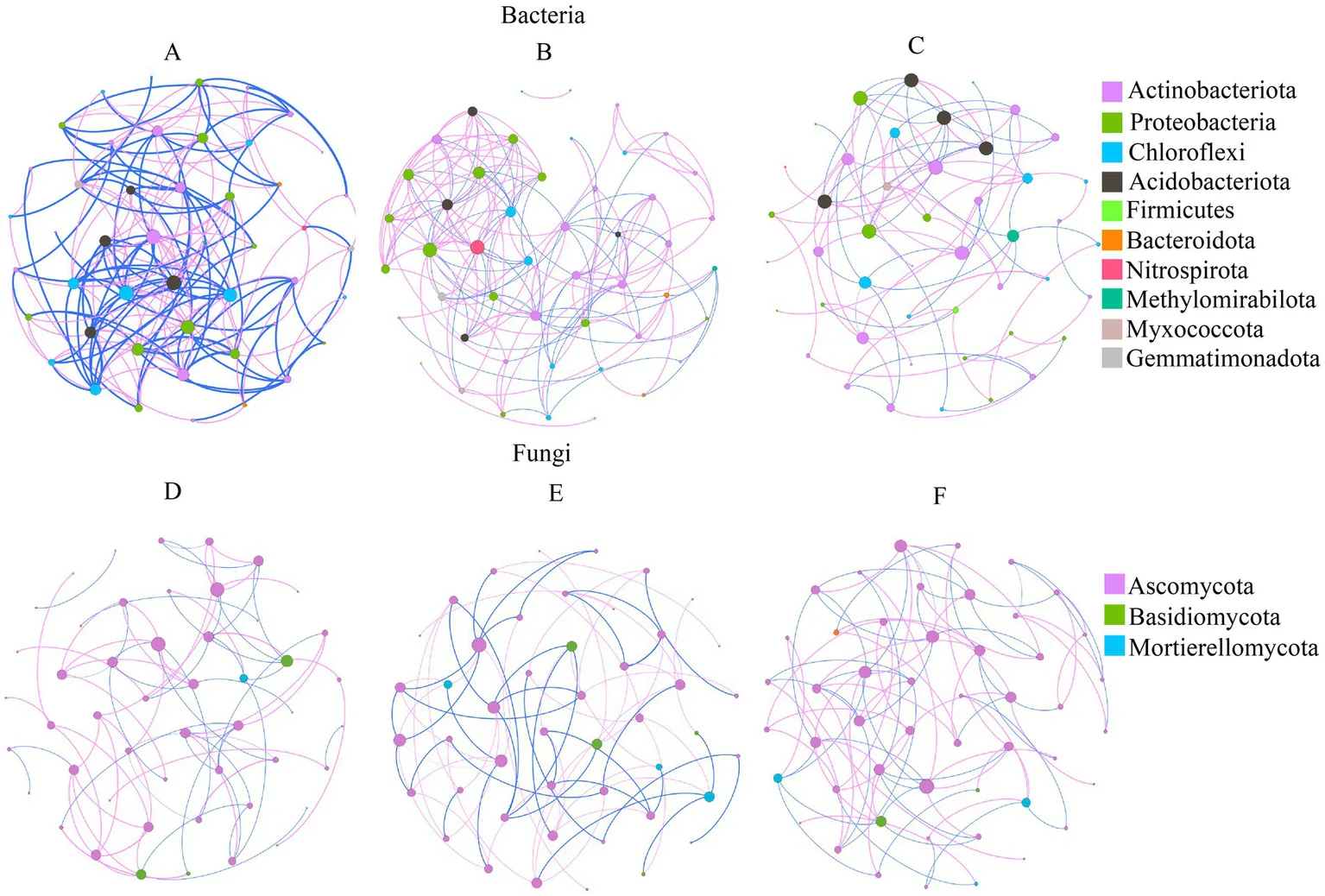
Co-occurrence networks of bacteria **(A–C)** and fungi **(D–F)** obtained by Spearman’s correlation analysis on the top 50 most abundant bacterial and fungal genera in the CH system **(A,D)**, the LJ system **(B,E)**, and the ZYHY system **(C,F)**.

**Figure 5:**
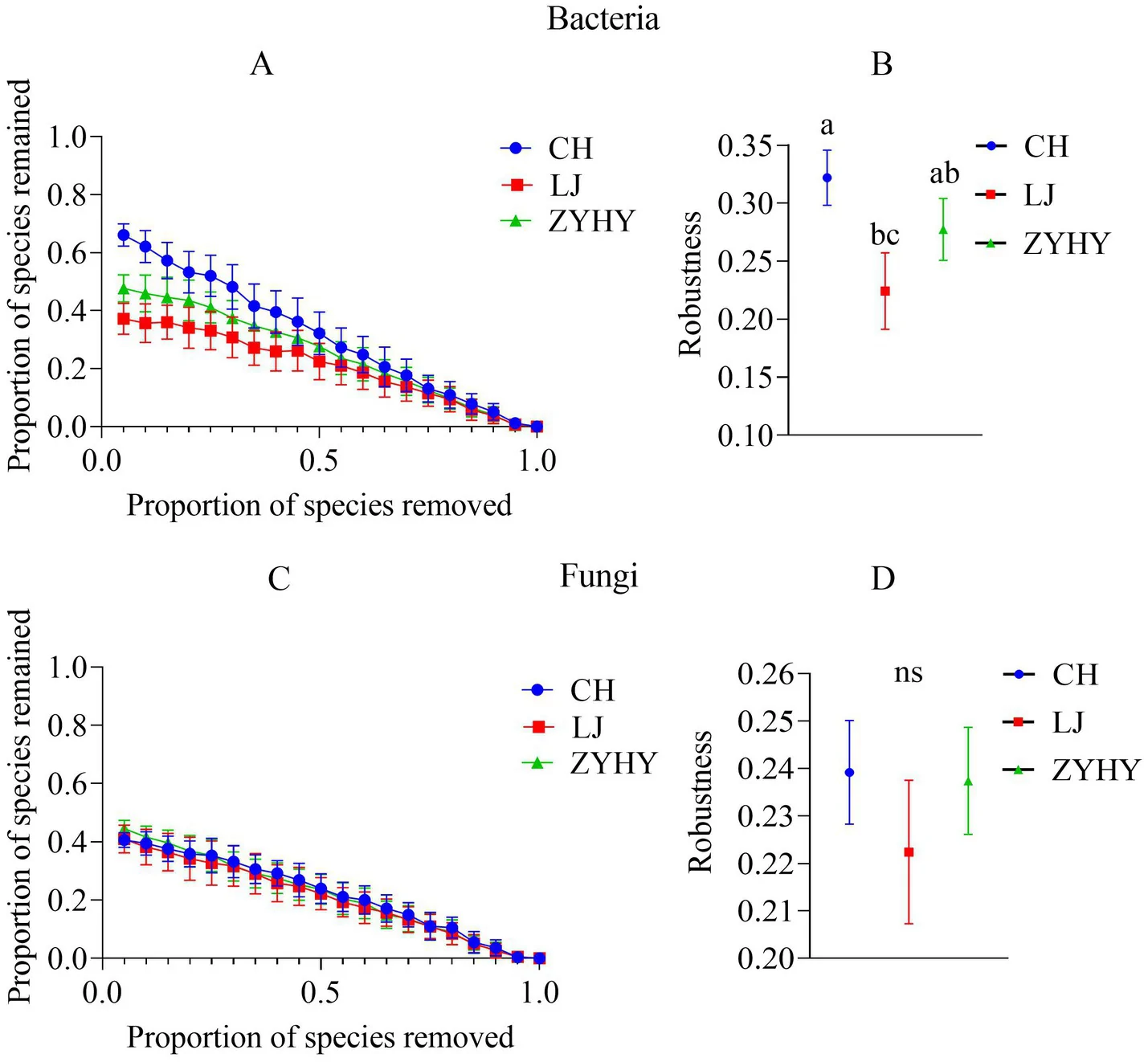
Stability of bacterial **(A,B)** and fungal networks **(C,D)** in safflower rhizosphere soils under different cultivation pattern-location combinations. Different lowercase letters indicate significant differences at *p* < 0.05. values were adjusted for multiple testing using the Benjamini–Hochberg (BH) method.

Comparisons were made among the sample-specific subnetworks extracted from the global co-occurrence networks of the top 50 most abundant bacterial and fungal genera across the CH, LJ, and ZYHY systems (Figure 4 and Table 3). For the bacterial subnetworks, the number of edges, average degree, graph density, and average clustering coefficient followed the order CH > LJ > ZYHY, while network diameter and average path length followed ZYHY > LJ and > CH. This pattern suggests that, within this analytical framework, the bacterial network in the CH system was the most complex and that in the ZYHY system the least complex. For the fungal subnetworks, the number of edges, average degree, graph density, and average clustering coefficient were highest in the ZYHY system, indicating the greatest complexity in this system under the same framework. Network stability was assessed using both robustness (based on the global networks) and the absolute ratio of negative to positive cohesion (based on sample subnetworks). Stability analysis (Figure 5 and Supplementary Figure S5) showed that bacterial network stability was highest in the CH system, whereas fungal network stability did not differ significantly among the three PLMEs. The observed complexity and stability patterns thus depend on the network-construction parameters and remain exploratory.

**Table 3:** Topological properties of sample-specific subnetworks.

Network	Node	Edge	Average degree	Graph density	Network diameter	Component	Modularity	Average clustering coefficient	Average path length
CH-B	47 ± 0.58a	190 ± 0.82a	8.08 ± 0.08a	0.18 ± 0.00a	8 ± 0.58d	1 ± 0.00d	0.37 ± 0.00e	0.57 ± 0.00a	2.89 ± 0.00f
LJ-B	48 ± 0.82a	185.16 ± 0.69b	7.71 ± 0.00b	0.16 ± 0.00b	8 ± 0.58d	3 ± 0.58a	0.45 ± 0.00d	0.51 ± 0.00b	3.16 ± 0.00e
ZYHY-B	45 ± 0.58b	88.83 ± 0.69c	3.96 ± 0.00c	0.09 ± 0.00c	13 ± 0.00b	1 ± 0.00d	0.61 ± 0.00c	0.39 ± 0.00e	4.89 ± 0.00b
CH-F	48 ± 0.94a	82.83 ± 1.57e	3.46 ± 0.06e	0.07 ± 0.00e	14.83 ± 0.69a	2 ± 0.00c	0.71 ± 0.00a	0.41 ± 0.00d	5.66 ± 0.01a
LJ-F	42 ± 1.29c	72 ± 0.82f	3.43 ± 0.00e	0.08 ± 0.00d	10 ± 0.00c	2 ± 0.00c	0.65 ± 0.00b	0.43 ± 0.00c	4.32 ± 0.00c
ZYHY-F	47 ± 0.00a	84.33 ± 0.47d	3.57 ± 0.00d	0.08 ± 0.00d	8 ± 0.00d	2.17 ± 0.00b	0.65 ± 0.00b	0.51 ± 0.00b	3.77 ± 0.00d

B, bacterial subnetwork; F, fungal subnetwork. Different lowercase letters indicate significant differences at *p* < 0.05. *p*-values were adjusted for multiple testing using the Benjamini–Hochberg (BH) method.

### 3.4 Composition and functional potential of rhizosphere soil microorganisms

**Figure 6:**
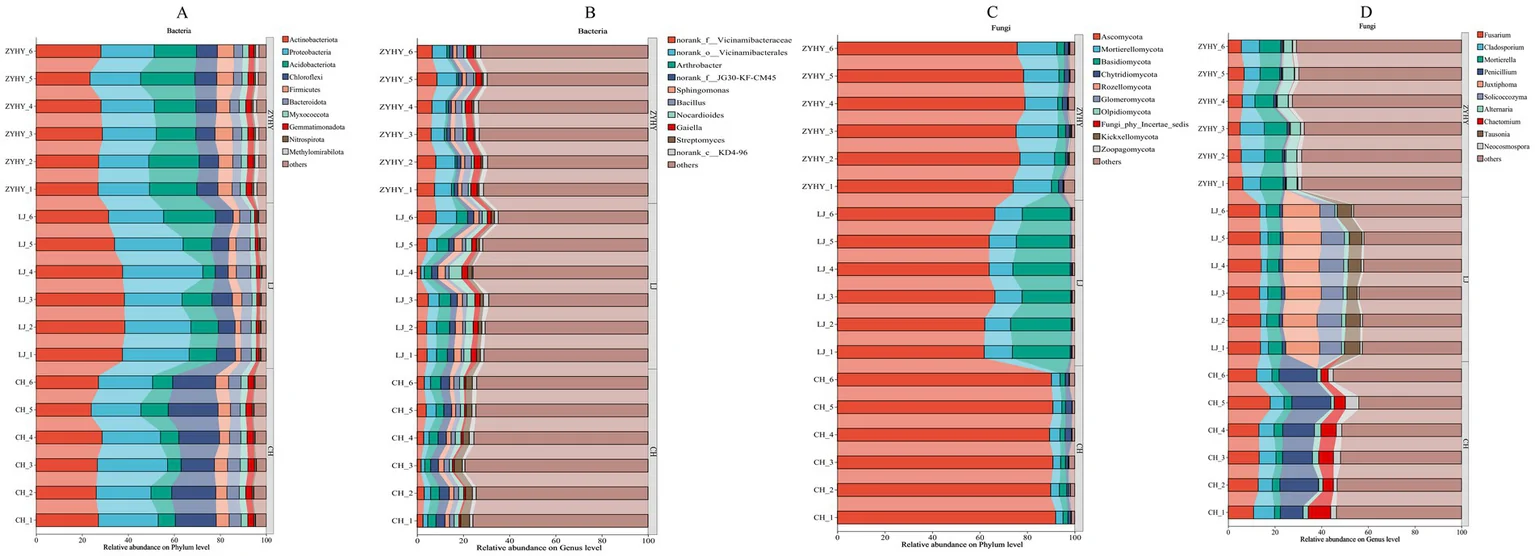
Bacterial **(A,B)** and fungal **(C,D)** community composition at the phylum **(A,C)** and genus **(B,D)** levels among safflower rhizosphere soils under different cultivation pattern–location combinations: CH (safflower rhizosphere soil with soybean as the preceding crop in Chenghai); LJ (safflower rhizosphere soil cultivated under apple orchards in Lijiang); ZYHY (safflower rhizosphere soil with tobacco as the preceding crop in Longpan).

The predominant bacterial phyla across all three rhizosphere soil samples were Actinobacteria, Proteobacteria, Acidobacteria, Chloroflexi, and Firmicutes, accounting for more than 80% of all bacterial phyla (Figure 6A). The CH samples had a higher relative abundance of Chloroflexi, the LJ samples had more Actinobacteria and Proteobacteria, and the ZYHY samples had more Acidobacteria and Firmicutes. The dominant bacterial genera were *Norank_f__Vicinamibacteraceae*, *Norank_o__Vicinamibacterales*, *Arthrobacter*, *Norank_f__JG30-KF-CM45*, and Sphingomonas, accounting for more than 10% of all bacterial genera (Figure 6B). Among these, *Norank_f__JG30-KF-CM45* was more abundant in CH samples, *Arthrobacter* and *Sphingomonas* were more abundant in LJ samples, and *Norank_f__Vicinamibacteraceae* and *Norank_o__Vicinamibacterales* were more abundant in ZYHY samples.

The leading fungal phyla were Ascomycota, Mortierellomycota, and Basidiomycota, accounting for over 90% of the total (Figure 6C). Among them, Ascomycota was more abundant in the CH samples, Basidiomycota was more abundant in the LJ samples, and Mortierellomycota was more abundant in the ZYHY samples. The dominant fungal genera were *Fusarium*, *Cladosporium*, *Mortierella*, *Penicillium*, and *Juxtiphoma* (Figure 6D). *Penicillium* was more abundant in CH samples, *Fusarium* and *Juxtiphoma* were more abundant in LJ samples, and *Cladosporium* and *Mortierella* were more abundant in ZYHY specimens.

In total, 23 bacterial phyla and 401 genera showed differences among the three PLMEs. In the CH system, 13 phyla and 187 genera were significantly enriched, including *Streptomyces*, *Steroidobacter*, and *Defluviicoccus*. In the LJ system, 3 phyla and 110 genera were significantly enriched, including *Nocardioides*, *Microvirga*, and *Blastococcus*. In the ZYHY system, 7 phyla and 104 genera were significantly enriched, including *Gaiella*, *Microlunatus*, and *Pedomicrobium*.

For fungi, 7 phyla and 130 genera showed differences among the three PLMEs. In the CH system, 1 phylum and 40 genera were significantly enriched, including *Chaetomium*, *Neocosmospora*, and *Aspergillus*. In the LJ system, 1 phylum and 30 genera were significantly enriched, including *Solicoccozyma*, *Linnemannia*, and *Plectosphaerella*. In the ZYHY system, 5 phyla and 60 genera were significantly enriched, including *Alternaria*, *Cumuliphoma*, and *Talaromyces*.

In addition, to account for the compositional nature of microbiome data, ALDEx2 analysis (FDR < 0.05, |effect size| > 1) was employed, revealing significant treatment-specific taxonomic shifts in both bacterial and fungal communities. In the bacterial community, genera such as *Defluviicoccus*, *Bryobacter*, and *Rhodococcus* were significantly enriched between the CH and LJ systems; *Candidatus Solibacter*, *Bacillus*, and *Gaiella* were associated with the divergence between CH and ZYHY systems; and further comparison between LJ and ZYHY systems revealed that taxa like *Microbacterium*, *Arthrobacter*, and *Clostridium sensu stricto* 1 were associated with the secondary succession between these two treatments (Supplementary Figure S1). Similarly, fungal communities exhibited strong responses: *Penicillium*, *Aspergillus*, and *Trichoderma* shifted significantly in the CH vs. LJ comparison; *Fusarium* and *Chaetomium* emerged as core differential taxa between CH and ZYHY systems; while in the direct comparison of LJ vs. ZYHY, genera such as *Juxtiphoma*, *Solicoccozyma*, and *Cephaliophora* displayed significant divergence (Supplementary Figure S2). Furthermore, global distribution and base abundance validations via Volcano and MA plots (Supplementary Figures S1, S2) confirmed that these top-ranked differential taxa possessed large effect sizes and high median abundances, successfully ruling out statistical artifacts from low-abundance sequencing noise.

### 3.5 Differences in rhizosphere soil microbial metabolism of safflower under different planting patterns

Based on LC–MS, 1,731 metabolites were identified from 18 soil samples, including lipids and lipid-like molecules (29.47%), organic heterocyclic compounds (16.17%), organic acids and derivatives (14.62%), benzenoids (11.30%), organic oxygen compounds (9.75%), phenylpropanoids and polyketides (7.46%), benzenoids (8.19%), nucleosides, nucleotides, and analogs (3.32%), organic nitrogen compounds (1.85%), alkaloids and derivatives (0.66%), lignans, neolignans, and related compounds (0.52%), hydrocarbon derivatives (0.22%), hydrocarbons (0.22%), organic sulfur compounds (0.15%), 1,3-dipoles (0.07%), and organohalogen compounds (0.07%), as well as some unclassified metabolites.

**Figure 7:**
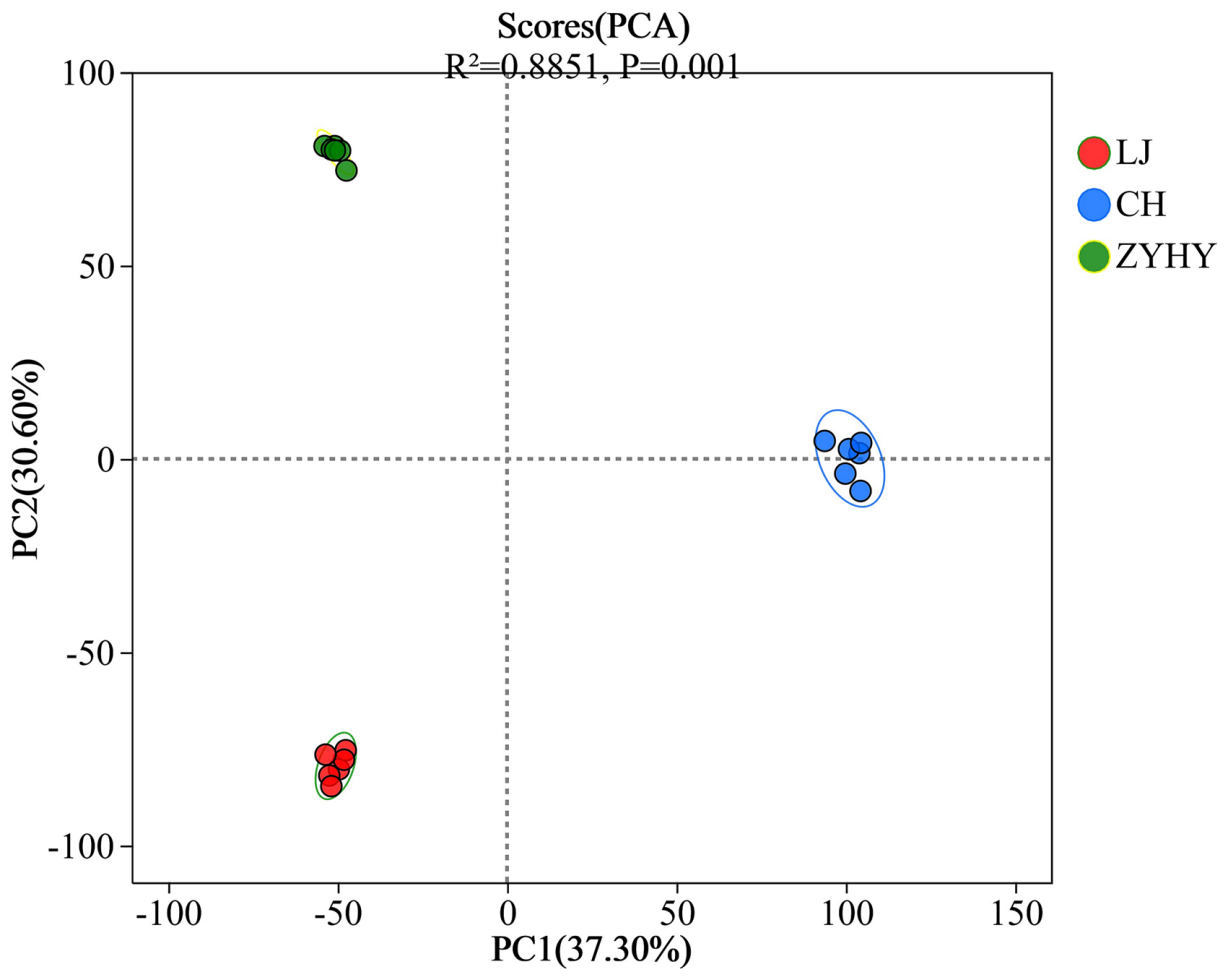
PCA of metabolites in safflower rhizosphere soil samples under different cultivation pattern-location combinations. Group differences were tested by PERMANOVA (999 permutations).

Principal component analysis (PCA) showed (Figure 7) that the metabolite compositions of CH, LJ, and ZYHY soils were significantly separated (*R*^2^ = 0.885, *p* = 0.001). A PERMDISP test was subsequently performed, and its *p*-value (0.058) exceeded 0.05, indicating that no statistically significant differences in multivariate dispersion were detected. Overall, 151 metabolites differed significantly among the three treatments. KEGG pathway enrichment analysis indicated that the differential metabolites (DMs) were mainly enriched in glycerophospholipid metabolism, arachidonic acid metabolism, cysteine and methionine metabolism, D-amino acid metabolism, and glycine, serine, and threonine metabolism.

**Figure 8:**
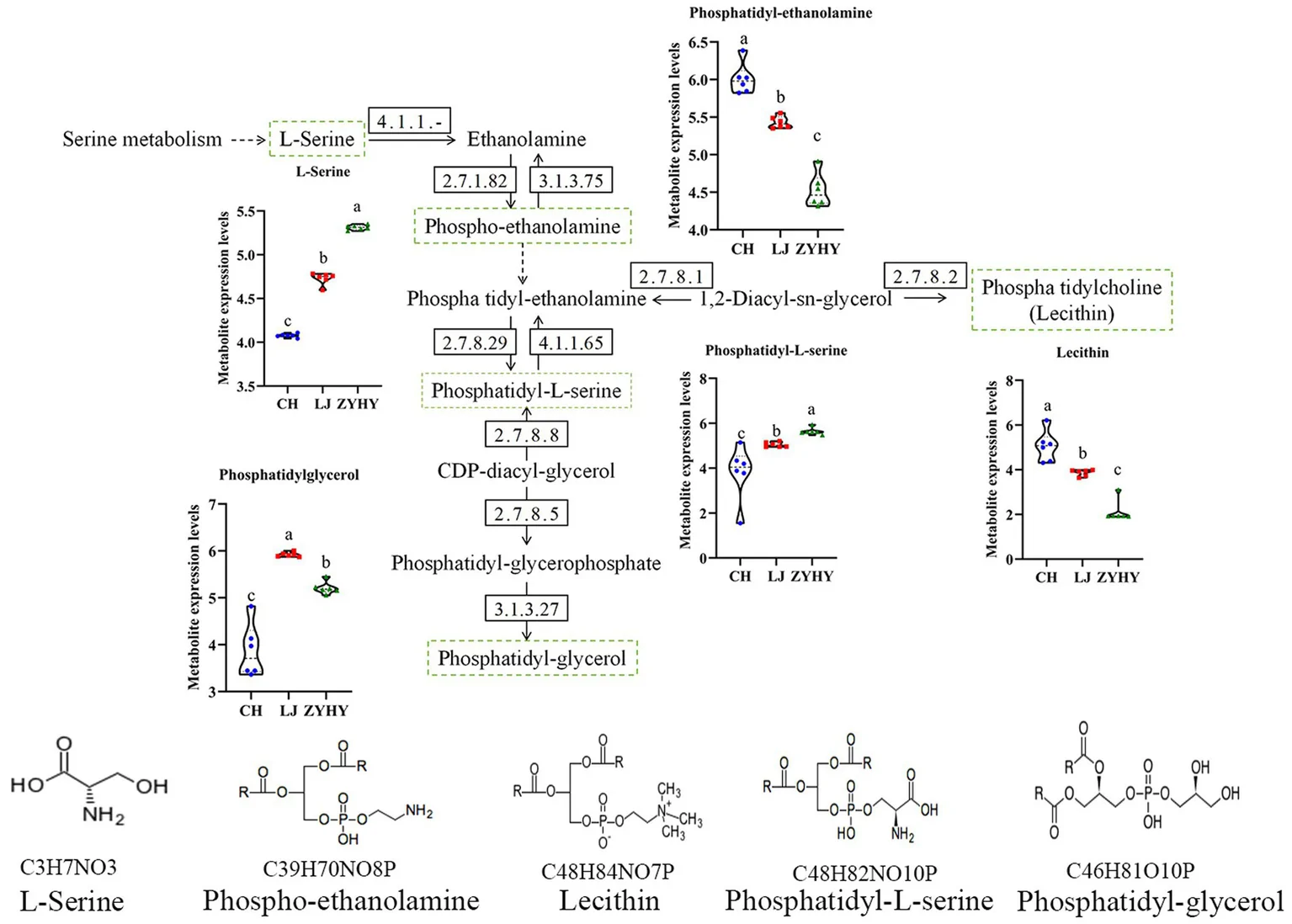
Differential metabolite profiles and molecular formulas of key differential metabolites in safflower rhizosphere soils under different cultivation pattern--location combinations.

The DMs among the three treatments were further screened, and revealed that they were mainly enriched in glycerophospholipid metabolism (ko00564). A metabolic pathway network involving five DMs was constructed (Figure 8), in which lecithin and phosphatidylethanolamine were most abundant in the CH system, L-serine and phosphatidyl-L-serine were most abundant in the ZYHY system, and phosphatidylglycerol was most abundant in the LJ system.

### 3.6 Relationship between soil metabolites and microorganisms

**Figure 9:**
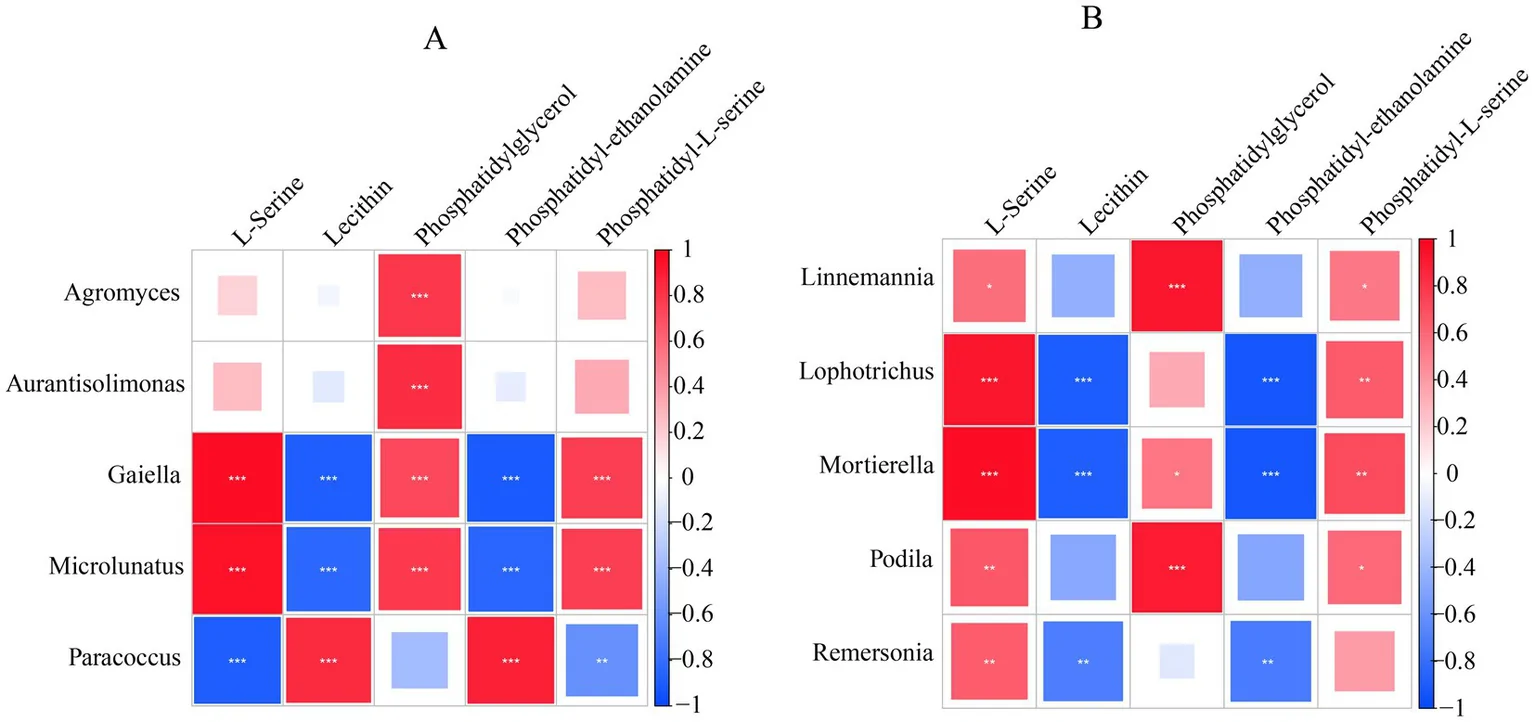
Correlation analysis between DMs **(A)** and differential bacterial **(A)** and fungal **(B)** genera within safflower rhizosphere soils under different cultivation pattern–location combinations **(B)**: CH (safflower rhizosphere soil with soybean as the preceding crop in Chenghai); LJ (safflower rhizosphere soil cultivated under apple orchards in Lijiang); ZYHY (safflower rhizosphere soil with tobacco as the preceding crop in Longpan). Red indicates a positive correlation, and blue indicates a negative correlation; darker colors represent stronger correlation coefficients and relationships; *p FDR < 0.05, **p _FDR_< 0.01, ***p _FDR_< 0.001. *P* values were adjusted for multiple testing using the Benjamini–Hochberg (BH) method.

Correlation analysis between the DMs enriched in the glycerophospholipid metabolism pathway and the differential microbial genera (bacterial and fungal) (Figure 9) showed that among bacterial genera, *Gaiella* and *Microlunatus* were significantly correlated with all metabolites, while among fungal genera, *Mortierella* was significantly correlated with all metabolites.

### 3.7 Relationships among soil physicochemical properties, microorganisms, and metabolites

**Figure 10:**
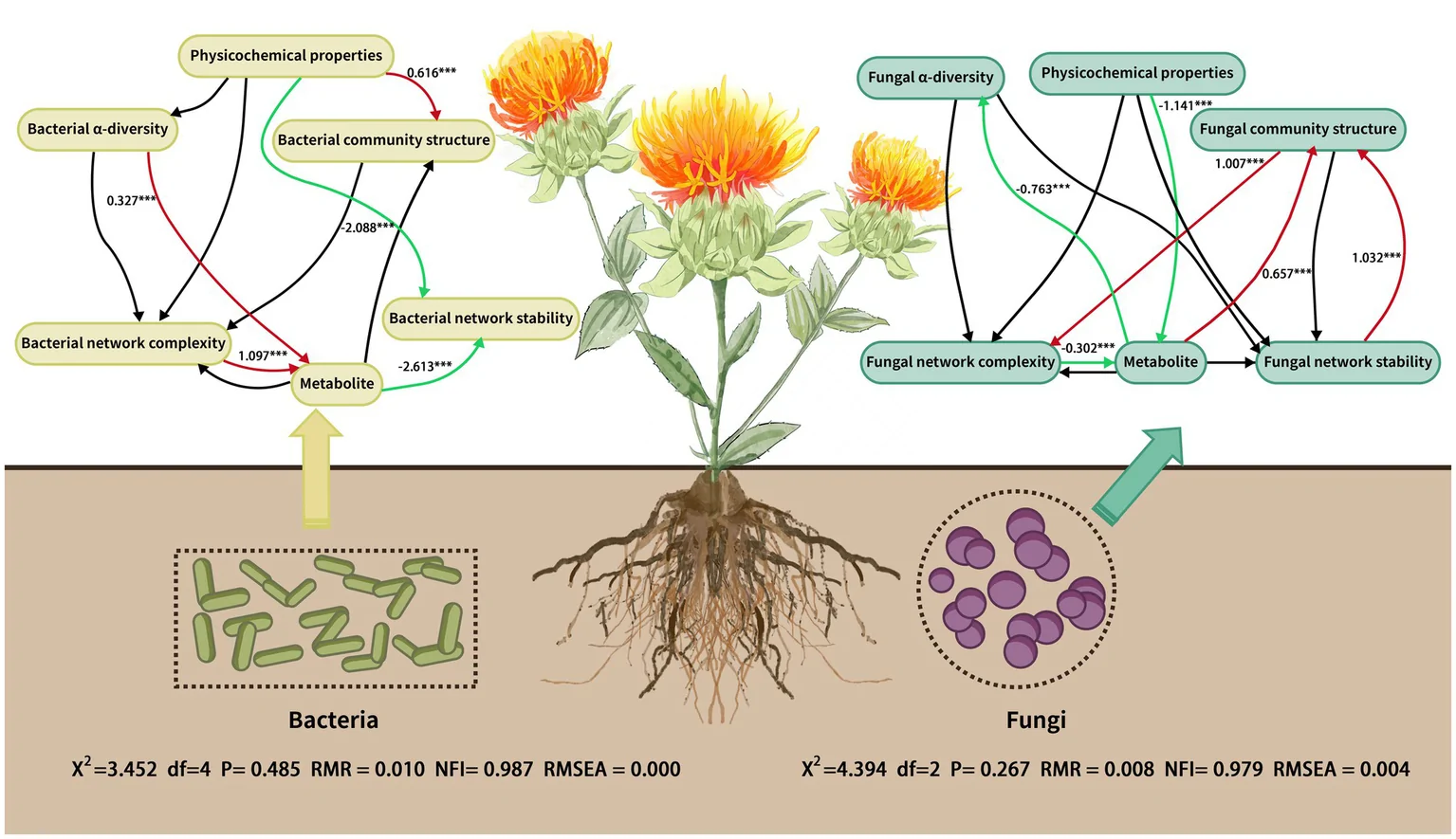
Exploratory SEM showing associations among soil physicochemical properties, microbial α-diversity (bacteria and fungi), microbial community structure, microbial network complexity, microbial network stability, and soil metabolites in safflower rhizosphere soils (*p < 0.05, ***p < 0.001). Green and red arrows indicate significant positive and negative associations, respectively; black arrows represent non-significant associations.

An exploratory structural equation modeling (SEM) analysis was performed to examine the potential correlations among soil physicochemical characteristics, microbial diversity (bacteria and fungi), microbial community structure, network complexity, network stability, and soil metabolites (Figure 10). This exploratory SEM suggested that bacterial α-diversity and network complexity were positively associated with soil metabolite composition, whereas fungal network complexity was negatively associated with metabolite composition.

## 4 Discussion

### 4.1 Diversity and community structure of bacteria and fungi in safflower rhizosphere soil differ among planting pattern-location combinations

Crop rotation and understory planting are intensive land-use cultivation practices that, by applying the principle of complementary ecological niches in compound communities (Sun et al., 2024), achieve high-yield, high-efficiency, and sustainable cropping systems. Therefore, they are widely used both domestically and internationally as potential strategies to resolve crop land competition. The associations of these practices are closely associated with variations in soils, which may be associated with the diversity and composition of soil microbial communities. Different cropping patterns and crop types have varying associations with soil microbial communities. Prior studies have indicated that both rotation and understory planting are associated with clear increases in soil microbial diversity indices (Liu et al., 2023). This might be correlated with increasing aboveground vegetation-associated biomass in compound communities, which was subsequently associated with microbial diversity. However, our findings indicate that microbial α-diversity of safflower rhizosphere soils is higher under rotation systems (CH and ZYHY) than in understory planting (LJ). This may be because alternating cultivation of different short-season crops in rotation systems is associated with greater variation in root exudates and residue types, which may provide more ecological niches and diversified nutrients for microbial proliferation (Qiao et al., 2024). Correlation analysis showed that HN, AP, and AK were most strongly and negatively associated with bacterial α-diversity, whereas EC (positive) and CAT activity (negative) showed the strongest correlations with fungal α-diversity. This suggests that lower levels of HN, AP, and AK – a result of the more efficient uptake and turnover of these nutrients in rotation systems due to diversified crop residues and root exudates-are associated with higher bacterial α-diversity. By contrast, fungal diversity is more dependent on the ionic environment reflected by soil EC and the oxidative metabolic status indicated by CAT activity. We speculate that by introducing diverse herbaceous crops, crop rotation is associated with increased nutrient mineralization (higher EC) and reduced oxidative stress (lower CAT activity). Rather than a simple enrichment of nutrients, these coordinated changes are associated with a more balanced and less competitive environment for microbial proliferation. However, the potential role of site-specific geographical conditions (e.g., altitude, climate, soil) in the higher α-diversity in rotation systems cannot be excluded.

This research found that the rhizosphere microbial community composition (bacteria and fungi) of safflower differed significantly among the three cultivation pattern–location combinations. This is partly consistent with previous studies on peanut rhizosphere bacterial community structure under different planting methods (peanut monoculture, peanut-rapeseed rotation, and peanut-maize intercropping followed by rapeseed rotation) (Cui et al., 2024). The differences might be associated with changes in root exudate composition and agricultural management practices under different combinations, as well as with site-specific factors, which are associated with soil physicochemical properties including pH, soil moisture, and soluble soil carbon, and subsequently with alterations in rhizosphere community structures (Wang et al., 2025; Sabir et al., 2021). Redundancy analysis (RDA) indicated that bacterial community structure was largely associated with EC and AK, whereas the fungal community was mainly associated with AP and EC. Soil salinity and ion strength, as reflected by EC, can be directly associated with bacterial osmotic balance and activity. AK is a readily available nutrient resource, affecting especially bacterial viability and environmental adaptability. In contrast, fungal communities may rely more on phosphorus supplied by AP. Therefore, we speculate that differences in microbial nutrient utilization strategies may be associated with the significant differences in safflower rhizosphere microbial community structure.

### 4.2 Planting patterns-location combinations are associated with the composition, network complexity, and stability of microbial communities in the safflower rhizosphere soil

The dominant bacterial phyla in safflower rhizosphere soils across the three cultivation patterns were Proteobacteria, Acidobacteria, Chloroflexi, and Firmicutes (accounting for over 80%, Figure 6A). The CH samples had a higher relative abundance of Chloroflexi; the LJ samples had higher proportions of Actinobacteria and Proteobacteria; and the ZYHY samples were enriched in Acidobacteria and Firmicutes. Many members of Actinobacteria and Proteobacteria are copiotrophs that are associated with soil nutrients, pathogen suppression, and plant growth (Javed et al., 2021; Spain et al., 2009). Many members of Acidobacteria (*Acidisarcina polymorpha* SBC82^T^, AB60) are known to utilize a variety of carbohydrates, and their relative abundance is positively correlated with the availability of organic carbon (Belova et al., 2022). Firmicutes, many members of which are considered copiotrophic, indicate that understory planting in Lijiang is associated with alterations in soil microbial composition, with increases in understory vegetation and shifts in the microbial community from adaptation to extremely nutrient-poor conditions toward a preference for nutrient-rich environments. Some Chloroflexi lineages can degrade complex organic matter (especially plant-derived matter such as cellulose) and are adapted to nutrient-poor soils. Additionally, certain Chloroflexi taxa can utilize more oxidized carbon forms, which may be associated with enhanced rhizosphere carbon sequestration, deposition, and transfer, and potentially with benefits for safflower growth (Niu et al., 2024). Certain Chloroflexi have also been linked to chloride degradation, potentially associated with alleviating nutrient stress on safflower growth (Liu et al., 2020).

The leading fungal phyla across the three groups were Ascomycota, Mortierellomycota, and Basidiomycota (comprising over 90%, Figure 6C). Among them, CH samples had a higher abundance of Ascomycota, the LJ samples had a higher abundance of Basidiomycota, while the ZYHY samples had a higher abundance of Mortierellomycota. Members of Ascomycota and Basidiomycota are key participants in carbon accumulation processes (Manici et al., 2024). Many Ascomycota species can efficiently degrade cellulose and hemicellulose (Miao et al., 2022). Representative Basidiomycota species, meanwhile, are more specialized in lignin degradation. Many Mortierellomycota taxa excel at decomposing complex organic materials that are difficult for other microbes to degrade, such as chitin, cellulose, and starch (Cui et al., 2025). By converting the fixed carbon and phosphorus into inorganic forms (e.g., phosphates) usable by plants and other soil microbes, these fungal groups are associated with the promotion of carbon and phosphorus cycles. This suggests that changes in rhizosphere fungal composition under different planting patterns may be associated with soil nutrient cycling.

However, soils from the three groups also showed some notable differences in fungal composition. *Chaetomium* was enriched in the CH system. It has been reported that many *Chaetomium* species exhibit strong decomposition activity and produce antimicrobial compounds, serving as important biocontrol resources that are associated with suppression of plant pathogens and mitigation of potential disease risks (Jiang et al., 2017). In the LJ system, enriched fungi include plant-pathogenic genera such as *Linnemannia* and *Plectosphaerella*. Taxa from these genera are associated with safflower root rot (Jiang et al., 2021). Simultaneously, *Solicoccozyma* was also significantly enriched in the LJ system. These results may reflect the disease pressure and microenvironmental stress associated with single-cropping safflower under apple understory. This may be associated with spatial distribution differences between apple and safflower roots in the soil. The ZYHY system enriched the largest number of fungal phyla (five phyla), exhibiting the most diverse functions, including pathogenic types (*Alternaria*) and beneficial rhizosphere microbes with biotechnological potential (*Talaromyces*), suggesting that tobacco rotation may be associated with a more complex and diverse soil fungal microecology, with specific associations requiring further evaluation based on species-level functions. These differences at the fungal community level reveal the potential associations of the three planting patterns–location combinations with soil health, plant growth, and disease risk.

Under the network-construction framework adopted in this study, the CH system displayed the highest bacterial network complexity and network stability, whereas the ZYHY system exhibited the highest fungal network complexity, with no significant differences in fungal network stability among systems. This pattern differs from previous studies that found that different planting patterns could be associated with significant changes in the complexity and stability of microbial networks in the rhizosphere of millet and cereals (Tian et al., 2022). One possible reason is that all three PLMEs in the study are reasonably well-adapted to local site conditions, and may be associated with a certain degree of rhizosphere microbial network homeostasis in safflower, albeit to different extents. Previous studies have demonstrated that network complexity is closely associated with soil health, nutrient cycling, and suppression of plant diseases; furthermore, healthy crop soils typically exhibit higher network complexity and stability (Shi et al., 2024; Zhang et al., 2021). The higher bacterial network complexity and stability observed in the CH system under our analytical conditions is therefore an interesting correlative signal. Whether it is associated with better plant performance requires further investigation.

### 4.3 Glycerophospholipid metabolism in safflower rhizosphere soil is significantly altered under different planting patterns-location combinations

Research has indicated that the composition of rhizosphere soil metabolites differs prominently under different planting patterns. As an illustration, legume-potato rotation was associated with significant increases in purine- and phenylalanine-related metabolites (Wang Y. et al., 2023); in perilla rhizosphere soils under different continuous cropping years, DMs correlated with lipid formation and oxidative stress processes (Liu Y. Q. et al., 2024), and so on. In our research, the safflower rhizosphere soil metabolites under the three planting patterns showed significant separation. KEGG pathway enrichment analysis indicated that these DMs were primarily enriched in glycerophospholipid metabolism, arachidonic acid metabolism, cysteine and methionine metabolism, D-amino acid metabolism, and glycine, serine, and threonine metabolism. The differential metabolites between the three treatments were further screened, revealing enrichment mainly in the glycerophospholipid metabolism pathway (ko00564). A metabolic pathway network involving five DMs was constructed (Figure 8), in which lecithin and phosphatidylethanolamine contents were highest in the CH system, L-serine and phosphatidyl-L-serine contents were highest in the ZYHY system, and phosphatidylglycerol content was highest in the LJ system.

Lipids are key players in plant membrane physiology, including modulation of hormone signaling and delivery, responses to abiotic stress, plasmodesmata function, and plant–microbe interplay. Classified as lipids, glycerophospholipids function as binding sites for intracellular and intercellular proteins while playing an important role in cell metabolism and signal transduction (Macabuhay et al., 2022). Glycerophospholipid metabolism is an important pathway in soil carbon cycling, and it is associated with the provision of organic carbon to the soil and with serving as a key response pathway for soil bacteria and fungi under environmental changes (Ding et al., 2020).

This research indicated that glycerophospholipid metabolites showed disparities in content among different planting patterns, likely associated with the characteristics of root exudates and their associations with the rhizosphere microenvironment. In the CH system, soybean as a nitrogen-fixing plant was associated with significantly increased soil nitrogen levels, and nitrogen is a key element for phospholipid synthesis; high nitrogen may be associated with enhanced microbial and plant root metabolic activity, which may be associated with membrane synthesis and renewal, and with the highest accumulation of lecithin and phosphatidylethanolamine. In the ZYHY system, secondary metabolites such as alkaloids released by tobacco may be associated with a “stress”-related condition in the rhizosphere microenvironment, and with metabolic responses that are associated with disease resistance and allelopathy. In the LJ system, shading by apple trees was associated with reduced understory light intensity; phosphatidylglycerol, as a key component of the photosynthetic membrane and stress response molecule, showed increased content, possibly reflecting that safflower and its rhizosphere microbes may be associated with adjustments in membrane lipid composition, which may be associated with optimization of light capture and utilization efficiency under low-light conditions, indicating that soil metabolic traits in this pattern are closely correlated with physiological adaptation to shading.

Although site-specific differences may have played a role to some extent, numerous studies have demonstrated that rhizosphere soil metabolite profiles are predominantly associated with plant growth status and neighboring plant-derived metabolites (Ulbrich et al., 2022; Wang Y. Z. et al., 2023). In our study, the observed variations in lipid contents may reflect specific biological processes associated with each planting pattern (e.g., nitrogen fixation, allelopathy, shading), which align well with known plant–plant interaction mechanisms. Therefore, in the above discussion, we interpret these metabolic variations as primarily associated with planting pattern differences arising from plant interspecific interactions, rather than with geographic confounding.

### 4.4 Planting patterns-location combinations may be associated with glycerophospholipid metabolism through different correlations with rhizosphere bacteria and fungi

The exploratory SEM results suggest that PLMEs show indirect associations with glycerophospholipid metabolites involving bacterial α-diversity, bacterial network complexity, and fungal network complexity. Specifically, bacterial α-diversity and network complexity showed positive correlations, whereas fungal network complexity showed a negative correlation with these metabolites. These association patterns may be associated with the specificity of rhizosphere microbe assembly associated with agricultural management and rhizosphere selection, which are jointly associated with the rhizosphere soil metabolic environment (Schmidt et al., 2019), and may indicate the key hub associations of microbial taxa in complex interactions.

Moreover, correlation analysis was performed between significantly enriched differential metabolites in the glycerophospholipid metabolism pathway and differential microbial genera (including bacteria and fungi) (Figure 9). It was found that *Gaiella, Microlunatus* and *Mortierella* were significantly correlated with all metabolites. Previous studies have shown that *Gaiella* and *Microlunatus* participate extensively in the decomposition of complex organic matter represented by lipids by secreting extracellular enzymes, and this participation is associated with changes in the structure and content of soil lipids (Wei et al., 2024). *Mortierella* can produce various polyunsaturated acids and is involved in cellulose and lignin degradation via extracellular enzymes, thus being associated with soil lipid decomposition (Li et al., 2023).

In summary, our exploratory analyses show that the abundances of *Gaiella*, *Microlunatus*, and *Mortierella* are associated with increased bacterial α-diversity, greater bacterial network complexity, and reduced fungal network complexity. We hypothesize that these microbial shifts may accompany glycerophospholipid metabolism in the safflower rhizosphere. This study provides an exploratory, correlative foundation for future hypothesis-driven experiments aimed at optimizing planting systems for safflower cultivation. In our follow-up work, we will further incorporate direct measurements of safflower growth, yield, quality, and disease incidence to more comprehensively validate and refine the above conclusions.

## 5 Conclusion

This research compared the rhizosphere soil microorganisms (bacteria and fungi) and their metabolic characteristics under three planting pattern–location combinations (CH, ZYHY, and LJ). Bacterial α-diversity was significantly higher under the CH system, while fungal α-diversity was highest under the ZYHY system. Bacterial α-diversity was strongly negatively correlated with soil AK, whereas fungal α-diversity was strongly negatively correlated with CAT activity. The rhizosphere microbial community composition significantly differed across the three treatments. Bacterial community variation was most strongly associated with EC, whereas fungal community variation was most closely associated with AP content. Based on the subnetworks analyzed, the CH bacterial network exhibited the highest topological complexity and stability, while the ZYHY system fungal network showed prominently higher complexity. Fungal network stability did not differ remarkably across treatments. These network metrics are inherently tied to the construction parameters and offer exploratory comparisons rather than absolute rankings. The relative abundance of dominant microbial phyla and genera markedly differed across treatments. Non-targeted metabolomics analysis revealed that glycerophospholipid metabolic pathways in the rhizosphere soil were significantly altered among treatments, and five key metabolites, including L-serine, phosphatidylethanolamine, and lecithin, were identified as biomarkers. Their abundance was strongly correlated with the abundance of *Gaiella*, *Microlunatus*, and *Mortierella*. Exploratory SEM further suggested that planting patterns–location combinations were indirectly associated with glycerophospholipid metabolism through associations with the rhizosphere microbiome, with bacterial α-diversity and network complexity showing significant positive associations, and fungal network complexity showing a significant negative association.

These correlative findings suggest that the CH system is associated with higher bacterial network complexity and stability, and with altered glycerophospholipid metabolism. Whether these microbial network patterns and metabolic differences are associated with improved safflower growth, yield, or disease resistance remains to be clarified. Future work will therefore integrate multi-season monitoring of key agronomic traits to further evaluate the functional significance of the observed microbiome and metabolome signatures.

## Data Availability

The original contributions presented in the study are included in the article/Supplementary material, further inquiries can be directed to the corresponding authors.

## Footnotes

1
https://www.ebi.ac.uk/metabolights
