# Supplementary material for: Microbiome network remodeling is associated with soil lipid metabolism in safflower under different cropping system-location combinations: an omics-based dissection
Source: Front Microbiol. 2026 Sep 7;17:1898896. doi: 10.3389/fmicb.2026.1898896 (PMC13590785; doi:10.3389/fmicb.2026.1898896)
Supplement: Supplementary file 1 [file Supplementary_file_1.DOCX]

Fig.S1 Differential abundance analysis of bacterial communities driven by different management practices.

(A) Lollipop plots showing the top 15 bacterial genera with the most significant differential abundance across pairwise comparisons (CH vs. LJ, CH vs. ZYHY, and LJ vs. ZYHY). The x-axis represents the ALDEx2 effect size. (B) Volcano plots illustrating the global differential abundance of bacterial taxa. Dashed lines indicate the significance thresholds (FDR < 0.05 and |Effect Size| > 1). (C) MA plots showing the relationship between median base abundance (log-ratio) and group differences. Across all panels, taxa significantly enriched in specific treatments are highlighted in red or blue, while non-significant taxa are shown in gray.

Fig.S2 Differential abundance analysis of fungal communities driven by different management practices.

(A) Lollipop plots showing the top 15 fungal genera with the most significant differential abundance across pairwise comparisons (CH vs. LJ, CH vs. ZYHY, and LJ vs. ZYHY). The x-axis represents the ALDEx2 effect size. (B) Volcano plots illustrating the global differential abundance of fungal taxa. Dashed lines indicate the significance thresholds (FDR < 0.05 and |Effect Size| > 1). (C) MA plots showing the relationship between median base abundance (log-ratio) and group differences. Across all panels, taxa significantly enriched in specific treatments are highlighted in red or blue, while non-significant taxa are shown in gray.

**Supplementary Material 2.1**

Soil pH was determined using a pH meter (Mettler Toledo, Switzerland) with a soil-to-water ratio of 2.5:1. Soil electrical conductivity (EC) was measured with a conductivity meter (DDB-303A, Shanghai INESA Scientific Instrument Co., Ltd.). Urease activity (SUE), alkaline phosphatase activity (ALP), and catalase activity (CAT) were determined using soil enzyme activity assay kits (Solarbio Technology Co., Ltd.)(Wang et al., 2025). Alkali-hydrolyzable nitrogen (HN) was measured using the alkali diffusion method. Ammonium nitrogen (NH_4_⁺-N) and nitrate nitrogen (NO₃⁻-N) were determined with a soil nutrient detector (HM-GT4, Shandong Hengmei Electronic Technology Co., Ltd.)(Wang et al., 2023). AP was measured by the molybdenum-antimony anti-colorimetric method, and available potassium (AK) was determined using the NH_4_OAc extraction-flame photometry method(Adams et al., 2025).

**Supplementary Material 2.2**

Microbial DNA was extracted from safflower rhizosphere soil samples using the E.Z.N.A. Soil DNA Kit (Omega Bio-Tek, Norcross, GA, USA) according to the manufacturer’s instructions, and DNA purity, concentration, and integrity were detected using a NanoDrop 2000(Liu et al., 2023). The V3-V4 region of the 16S rRNA gene was amplified using primers 338F (5′-ACTCCTACGGGAGGCAGCAG-3′) and 806R (5′-GGACTACHVGGGTWTCTAAT-3′). The fungal ITS sequence was amplified using primers ITS1F (5′-CTTGGTCATTTAGAGGAAGTAA-3′) and LR3 (5′-CCGTGTTTCAAGACGGG-3′). The PCR reaction system was: 4 μL 5× TransStart FastPfu buffer, 2 μL 2.5 mM dNTPs, 0.8 μL forward primer (5 μM), 0.8 μL reverse primer (5 μM), 0.4 μL TransStart FastPfu DNA polymerase, 10 ng template DNA, and nuclease-free water to a total volume of 20 μL. The amplification program was as follows: 95 °C pre-denaturation for 3 min; 27 cycles of 95 °C denaturation for 30 s, 55 °C annealing for 30 s, and 72 °C extension for 30 s; 72 °C final extension for 10 min; and storage at 4 °C (PCR instrument: ABI GeneAmp® 9700). During the amplification process, sterile water was used as a negative control and processed simultaneously with the samples. The absence of bands in the negative control indicated that no reagent or environmental contamination was introduced (Fig.S3). PCR products were recovered using 2% agarose gel electrophoresis, purified with a DNA Gel Extraction Kit (PCR Clean-Up Kit, YuHua, China), and quantified using Qubit 4.0 (Thermo Fisher Scientific, USA).

Bacterial 16S rRNA and fungal ITS fragments were sequenced on the Illumina MiSeq PE300 platform. After PCR product identification and purification, the MiSeq library was constructed using the NEXTFLEX® Rapid DNA-Seq Kit (Bioo Scientific, Austin, TX, USA). Raw sequences were quality-controlled with Trimmomatic 0.32 (Edger et al., 2019), spliced with FLASH 1.2.11 (Magoc and Salzberg, 2011) and UPARSE 7.1 (Edgar, 2013), and clustered into OTUs at 97% similarity (Stackebrandt et al., 1994) using the USEARCH algorithm. Chimeras were removed with UCHIME 4.1 (Edgar et al., 2011) to obtain the OTU table. Each sequence was taxonomically annotated with the RDP classifier (http://rdp.cme.msu.edu/, Gao et al., 2017). Bacterial sequences were compared with the Silva database (SSU128, Henderson et al., 2019), and fungal sequences with the Unite database (version 8.0; http://unite.ut.ee/index.php, Abarenkov et al., 2010), with a classification threshold of 70%. After taxonomic filtering and rarefaction to the minimum sequencing depth (“vegan” package in R 4.1.0), the processed data were used for downstream analysis. The filtering criteria were as follows: (i) OTUs were retained only if they had more than 5 sequences in at least 3 samples; (ii) OTUs with a total sequence count greater than 20 were retained; (iii) OTUs with a relative abundance less than 0.01% were removed; (iv) OTUs derived from chloroplasts and mitochondria were also removed. The rarefaction depths for 16S rRNA and ITS sequencing were 45,518 and 54,952, respectively. The average trimmed sequence lengths were 243 bp for 16S rRNA and 376 bp for ITS. The sequences have been uploaded to the NCBI Sequence Read Archive under accession numbers (16S rRNA data) and (ITS data).

Fig.S3 Agarose gel electrophoresis of PCR-amplified (a) 16S rRNA and (b) ITS fragments from all samples.

Table S1 Raw sequence counts per sample for bacterial 16S and fungal ITS.

| Sample Name | Raw sequence counts of 16S rRNA sequencing | Raw sequence counts of ITS sequencing |
| --- | --- | --- |
| CH1 | 60028 | 84945 |
| CH2 | 69022 | 73062 |
| CH3 | 59760 | 79610 |
| CH4 | 66813 | 67845 |
| CH5 | 61678 | 67443 |
| CH6 | 60092 | 68630 |
| LJ1 | 51046 | 75008 |
| LJ2 | 49938 | 78345 |
| LJ3 | 54334 | 71146 |
| LJ4 | 50370 | 71828 |
| LJ5 | 55312 | 68302 |
| LJ6 | 50534 | 63941 |
| ZYHY1 | 56003 | 58885 |
| ZYHY2 | 55559 | 62610 |
| ZYHY3 | 54264 | 61109 |
| ZYHY4 | 57042 | 65413 |
| ZYHY5 | 62595 | 56704 |
| ZYHY6 | 49033 | 65085 |

**Supplementary Material 2.3**

One gram of fresh soil was mixed with 1 mL of extraction solution (methanol:water = 4:1, v/v) and ground for 6 min in a cryogenic tissue grinder (-10 °C, 50 Hz), followed by low-temperature ultrasonic extraction for 30 min (5 °C, 40 kHz). The sample solution was then kept at -20 °C for 30 min, followed by low-temperature centrifugation for 15 min (4 °C, 13,000 g). The obtained supernatant was used for detection. LC-MS/MS analysis was performed using a UHPLC-Q Exactive HF-X system. The Acquity UPLC HSS T3 column (100 mm × 2.1 mm, 1.8 μm; Waters, Milford, USA) was used with a 3 μL injection volume and 40°C column temperature. The mobile phases A and B used for elution were 95% water + 5% acetonitrile (containing 0.1% formic acid) and 47.5% acetonitrile + 47.5% isopropanol + 5% water (containing 0.1% formic acid), respectively. QC samples were prepared by pooling equal volumes of all sample extracts to match the volume of individual experimental samples. One QC sample was injected every 5–15 analytical runs to monitor analytical stability. A pure water blank was injected at the start of the sequence to monitor system background and carryover.

The raw data were first processed with Progenesis QI software (Waters Corporation, Milford, USA) to generate a data matrix containing sample name, m/z, retention time, and peak intensity. Before further analysis, features with >20% missing values per group in the raw data were removed. Missing values were imputed with the global minimum, and peak intensities were sum-normalized. Variables with RSD >30% in QC samples were excluded, and data were log₁₀-transformed to yield the final matrix. Metabolite identification was performed by matching the precursor m/z within 5 ppm mass accuracy and MS/MS fragmentation patterns against public databases including HMDB (v5.0) and Metlin, as well as the in-house Majorbio database (MJDB). All 1,731 metabolites were identified at Level 2 (secondary MS matching); 836 (48.29%) were further confirmed at Level 1 using reference standards. No lower-level identifications were included.

To preliminarily assess inter-group metabolic differences and intra-group variability, PCA (using ropls v1.6.2 in R) was performed on all samples including QCs. PC1, PC2, and PC3 explained 35.3%, 29.2%, and 5.4% of total variance, respectively (Fig. S4, P < 0.001). QC samples clustered tightly, confirming analytical stability and reproducibility. Differential metabolites were selected based on the following criteria: (i) Variable Importance in the Projection (VIP) > 1 obtained from the OPLS-DA model (one orthogonal component). (ii) Raw p-value < 0.05 from Student’s t-test, and (iii) absolute fold change (FC) ≧ 1.2 or ≦ 0.83. To control the false discovery rate (FDR), the p-values were further adjusted using the Benjamini–Hochberg method, and metabolites with adjusted p-value < 0.05 were considered statistically significant. Metabolic pathway enrichment was evaluated using a hypergeometric test implemented in Python (scipy v1.0.0; <https://scipy.org>). The background set comprised all identified metabolites in the study. The KEGG pathway annotations for each metabolite were obtained from the KEGG API. *P* values were adjusted using the Benjamini–Hochberg procedure. Pathways with adjusted p < 0.05 were considered significantly enriched.

Fig.S4 PCA score plot of metabolites in safflower rhizosphere soil samples under different treatments, with QC samples included (LJ, safflower rhizosphere soil cultivated under apple orchards in Lijiang; CH, safflower rhizosphere soil with soybean as the preceding crop in Chenghai; ZYHY, safflower rhizosphere soil with tobacco as the preceding crop in Longpan).

**Supplementary Material 2.4**

For each system, genus-level relative abundance tables were filtered to retain only genera present in ≥4 of 6 biological replicates (≥66.7% prevalence) and with mean relative abundance ≥0.1%. From these, the top 50 most abundant bacterial and fungal genera were selected for network construction (Banerjee et al., 2016). To address compositionality, zeros were replaced using Bayesian-multiplicative replacement (zCompositions::cmultRepl, threshold = 0.65), followed by group-wise centered log-ratio (CLR) transformation. Spearman correlation coefficients (psych R package) were calculated, and only edges with |ρ| > 0.70 and Benjamini–Hochberg–adjusted p < 0.05 were retained. Thus, for each of the three systems (CH, LJ, ZYHY), one global co‑occurrence network was generated for bacteria and one for fungi, based solely on the samples from that system.

For sample-level analysis, subnetworks were extracted (igraph R package) containing only genera detected in each sample and the edges among them inherited from the system-specific global network. Topological coefficients of each sample subnetwork (node number, edge number, average degree, graph density, network diameter, component, modularity, average clustering coefficient and average path length) were calculated to estimate network complexity. Network visualization is presented using the Gephi v0.9.2 software. Generally, larger values of the number of nodes, number of edges, average degree, graph density, and average clustering coefficient, along with smaller values of average path length and graph diameter, indicate a more complex network. To assess how random node removal affects network stability, nodes were removed from 5% to 100% and the proportion of remaining nodes were measured (100 random replicates per rate). At 50% removal, the mean remaining node proportion was compared (Robustness) via permutation-based significance testing (p＜0.05). A higher proportion indicates greater stability (de Vries et al., 2018; Zhang et al., 2021). As a complementary stability measure, positive and negative cohesion were computed for the subnetworks (six per sample) using igraph, and the absolute ratio of negative to positive cohesion was used as an additional indicator of network stability. A higher absolute ratio indicates greater network stability (de Vries et al., 2018).

All subnetwork‑level metrics reported in this study depend on the thresholds and procedures adopted for global network construction. These descriptors should therefore be interpreted as exploratory, as alternative parameter choices may yield different topological patterns.

**Supplementary Material 2.5**

Microbial community analysis was conducted using Mothur (v1.30.2) to calculate the Shannon and Chao1 indices for evaluating bacterial and fungal α-diversity. Differences in α-diversity and soil physicochemical properties among the three planting patterns (CH, LJ, ZYHY) were assessed by one-way ANOVA, followed by Tukey’s HSD test if significant. Normality (Shapiro–Wilk) and homogeneity of variance (Levene’s test) were checked prior to ANOVA. If assumptions were violated, Box–Cox transformation was applied.

Based on the OTU abundance table (97% similarity), Principal coordinate analysis (PCoA, vegan package in R v.3.3.1) was used for ordination. Permutational multivariate analysis of variance (PERMANOVA, ggplot2 package in R v.3.3.1, adonis function with 999 permutations) was performed to test for significant differences in community structure among groups (Li et al., 2022). The homogeneity of multivariate dispersions among groups was evaluated with a permutation test of multivariate dispersions (PERMDISP) using the ‘betadisper’ function in vegan (999 permutations). This step was performed to verify that significant PERMANOVA results were not driven primarily by differences in within-group dispersion.

Community composition bar plots were generated using the barplot package. LEfSe (LDA score > 2, p < 0.05) identified OTUs with significantly different abundances among groups. DESeq2 (negative binomial model, FDR-corrected p<0.05) was used for independent validation.

Pearson correlation analysis (Hmisc package in R v.3.3.1) was used to examine the relationships between soil physicochemical properties, microbial α-diversity, and metabolites. p values were FDR-corrected. Significant correlations (p< 0.05) were visualized in TBtools.

RDA (Canoco 5) was employed to analyze the influence of soil physicochemical properties on microbial β-diversity. Significantly different metabolites (detailed in Supplementary Material 2.3, analyzed with the R ropls package) were used for PCA and volcano plot visualization. KEGG pathway enrichment analysis was performed using Fisher's exact test (scipy package), with FDR correction (Benjamini-Hochberg); pathways with FDR<0.05 were considered significantly enriched. Metabolic network maps were illustrated with Adobe Illustrator CC 2019.

An exploratory structural equation model (SEM) was constructed in SPSS Amos (v 26.0) to examine the potential associations among soil physicochemical properties, microbial α-diversity, community structure, network stability, network complexity, and metabolites in safflower rhizosphere soils under three planting pattern–location combinations. Model fit was evaluated based on approximate fit indices (p>0.05), RMSEA/RMR (approaching 0), and GFI/NFI (approaching 1) (Yu et al., 2020). It should be noted that the model is intended for exploratory hypothesis generation rather than confirmatory causal inference; all reported paths are interpreted as associations.

**References**

Abarenkov, K., Nilsson, R.H., Larsson, K.H., Alexander, I.J., Eberhardt, U., Erland, S., Hoiland, K., Kjoller, R., Larsson, E., Pennanen, T., Sen, R., Taylor, A.F.S., Tedersoo, L., Ursing, B.M., Vrålstad, T., Liimatainen, K., Peintner, U., Koljalg, U., 2010. The UNITE database for molecular identification of fungi - recent updates and future perspectives. New Phytol. 186 (2), 281-285. <https://doi.org/10.1111/j.1469-8137.2009.03160.x>.

Adams, Z., Modi, A.T., Kuria, S.K., 2025. Multidimensional Perspective of Sustainable Agroecosystems and the Impact on Crop Production: A Review. Agriculture-Basel 15 (6), 26. <https://doi.org/10.3390/agriculture15060581>.

Banerjee, S., Kirkby, C.A., Schmutter, D., Bissett, A., Kirkegaard, J.A., Richardson, A.E., 2016. Network analysis reveals functional redundancy and keystone taxa amongst bacterial and fungal communities during organic matter decomposition in an arable soil. Soil Biol. Biochem. 97, 188-198. <https://doi.org/10.1016/j.soilbio.2016.03.017>.

de Vries, F.T., Griffiths, R.I., Bailey, M., Craig, H., Girlanda, M., Gweon, H.S., Hallin, S., Kaisermann, A., Keith, A.M., Kretzschmar, M., Lemanceau, P., Lumini, E., Mason, K.E., Oliver, A., Ostle, N., Prosser, J.I., Thion, C., Thomson, B., Bardgett, R.D., 2018. Soil bacterial networks are less stable under drought than fungal networks. Nature Communications 9, 12. <https://doi.org/10.1038/s41467-018-05516-7>.

Edgar, R.C., 2013. UPARSE: highly accurate OTU sequences from microbial amplicon reads. Nat. Methods 10 (10), 996-+. <https://doi.org/10.1038/nmeth.2604>.

Edgar, R.C., Haas, B.J., Clemente, J.C., Quince, C., Knight, R., 2011. UCHIME improves sensitivity and speed of chimera detection. Bioinformatics 27 (16), 2194-2200. <https://doi.org/10.1093/bioinformatics/btr381>.

Edger, P.P., Poorten, T., VanBuren, R., Hardigan, M.A., Colle, M., McKain, M.R., Smith, R.D., Teresi, S., Nelson, A.D.L., Wai, C.M., Alger, E., Bird, K.A., Yocca, A.E., Pumplin, N., Ou, S.J., Ben-Zvi, G., Brode, A., Baruch, K., Swale, T., Shiue, L., Acharya, C.B., Cole, G.S., Mower, J.P., Childs, K.L., Jiang, N., Lyons, E., Freeling, M., Puzey, J.R., Knapp, S.J., 2019. Origin and evolution of the octoploid strawberry genome. Nat. Genet. 51 (3), 541-+. <https://doi.org/10.1038/s41588-019-0356-4>.

Gao, X., Lin, H.Y., Revanna, K., Dong, Q.F., 2017. A Bayesian taxonomic classification method for 16S rRNA gene sequences with improved species-level accuracy. BMC Bioinformatics 18, 10. <https://doi.org/10.1186/s12859-017-1670-4>.

Henderson, G., Yilmaz, P., Kumar, S., Forster, R.J., Kelly, W.J., Leahy, S.C., Guan, L.L., Janssen, P.H., 2019. Improved taxonomic assignment of rumen bacterial 16S rRNA sequences using a revised SILVA taxonomic framework. PeerJ 7, 18. <https://doi.org/10.7717/peerj.6496>.

Li, M., Zhang, K.R., Yan, Z.Q., Liu, L., Kang, E.Z., Kang, X.M., 2022. Soil Water Content Shapes Microbial Community Along Gradients of Wetland Degradation on the Tibetan Plateau. Front. Microbiol. 13, 10. <https://doi.org/10.3389/fmicb.2022.824267>.

Liu, J.X., Jiang, H.D., Huang, Y., Zhong, L.S., Xu, Q., Yang, Q.G., Liu, S.Y., Wei, X., Liang, Y., Chai, S.F., 2023. Combined analysis of metagenome and transcriptome revealed the adaptive mechanism of different golden Camellia species in karst regions. Front. Plant Sci. 14, 15. <https://doi.org/10.3389/fpls.2023.1180472>.

Magoc, T., Salzberg, S.L., 2011. FLASH: fast length adjustment of short reads to improve genome assemblies. Bioinformatics 27 (21), 2957-2963. <https://doi.org/10.1093/bioinformatics/btr507>.

Stackebrandt, E., Goebel, B. M. (1994). Taxonomic note: a place for DNA-DNA reassociation and 16S rRNA sequence analysis in the present species definition in bacteriology. International journal of systematic and evolutionary microbiology, 44(4), 846-849. https://doi.org/10.1099/00207713-44-4-846

Wang, J., Wang, Y.Y., Tan, W., Zhao, D., Xu, C.M., Wang, Z.W., Liu, Y.F., Liu, S.Y., Kang, J.Y., 2025. Study on the Ecological Interaction Mechanism of Continuous Cropping Soil Driven by Different Modifiers. Horticulturae 11 (5), 18. <https://doi.org/10.3390/horticulturae11050446>.

Wang, X.Y., Wang, M.M., Chen, L., Shutes, B., Yan, B.X., Zhang, F.M., Lyu, J., Zhu, H., 2023. Nitrogen migration and transformation in a saline-alkali paddy ecosystem with application of different nitrogen fertilizers. Environ. Sci. Pollut. Res. 30 (18), 51665-51678. <https://doi.org/10.1007/s11356-023-25984-9>.

Yu, F.M., Lin, J.M., Xie, D.Y., Yao, Y.W., Wang, X.R., Huang, Y.Y., Xin, M.F., Yang, F.F., Liu, K.H., Li, Y., 2020. Soil properties and heavy metal concentrations affect the composition and diversity of the diazotrophs communities associated with different land use types in a mining area. Applied Soil Ecology 155, 9. <https://doi.org/10.1016/j.apsoil.2020.103669>.

Zhang, L.Y., Delgado-Baquerizo, M., Shi, Y., Liu, X., Yang, Y.F., Chu, H.Y., 2021. Co-existing water and sediment bacteria are driven by contrasting environmental factors across glacier-fed aquatic systems. Water Res. 198, 12. <https://doi.org/10.1016/j.watres.2021.117139>.

Table S2 Permutest results of multivariate homogeneity of group dispersions (PERMDISP) for bacterial and fungal communities.

|  | Df | Sum Sq | Mean Sq | F | N.Perm | Pr(>F) |
| --- | --- | --- | --- | --- | --- | --- |
| 16S rRNA | 2 | 0.022 | 0.001 | 1.191 | 999 | 0.351 |
| Residuals | 15 | 0.014 | 0.001 |  |  |  |
| ITS | 2 | 0.002 | 0.001 | 1.190 | 999 | 0.319 |
| Residuals | 15 | 0.014 | 0.001 |  |  |  |

Fig.S5 Cohesion absolute values for bacterial (A) and fungal (B) subnetworks.
